# The environment as a driver of immune and endocrine responses in dolphins (*Tursiops truncatus*)

**DOI:** 10.1371/journal.pone.0176202

**Published:** 2017-05-03

**Authors:** Patricia A. Fair, Adam M. Schaefer, Dorian S. Houser, Gregory D. Bossart, Tracy A. Romano, Cory D. Champagne, Jeffrey L. Stott, Charles D. Rice, Natasha White, John S. Reif

**Affiliations:** 1 National Oceanic and Atmospheric Administration, National Ocean Service, Center for Coastal Environmental Health & Biomolecular Research, Charleston, SC, United States of America; 2 Department of Public Health Sciences, Medical University of South Carolina, Charleston, SC, United States of America; 3 Harbor Branch Oceanographic Institution at Florida Atlantic University, Ft. Pierce, FL, United States of America; 4 Marine Mammal Foundation, San Diego, CA, United States of America; 5 Georgia Aquarium, Atlanta, GA, United States of America; 6 Division of Comparative Pathology, Miller School of Medicine, University of Miami, Miami, FL, United States of America; 7 Mystic Aquarium, a division of Sea Research Foundation, Mystic, CT, United States of America; 8 University of California, Davis, United States of America; 9 Department of Biological Sciences, Graduate Program in Environmental Toxicology, Clemson University, Clemson, SC, United States of America; 10 Department of Environmental and Radiological Health Sciences, Colorado State University, Fort Collins, CO, United States of America; Glasgow Caledonian University, UNITED KINGDOM

## Abstract

Immune and endocrine responses play a critical role in allowing animals to adjust to environmental perturbations. We measured immune and endocrine related markers in multiple samples from individuals from two managed-care care dolphin groups (n = 82 samples from 17 dolphins and single samples collected from two wild dolphin populations: Indian River Lagoon, (IRL) FL (n = 26); and Charleston, (CHS) SC (n = 19). The immune systems of wild dolphins were more upregulated than those of managed-care-dolphins as shown by higher concentrations of IgG and increases in lysozyme, NK cell function, pathogen antibody titers and leukocyte cytokine transcript levels. Collectively, managed-care care dolphins had significantly lower levels of transcripts encoding pro-inflammatory cytokine TNF, anti-viral MX1 and INFα and regulatory IL-10. IL-2Rα and CD69, markers of lymphocyte activation, were both lower in managed-care care dolphins. IL-4, a cytokine associated with T_H_2 activity, was lower in managed-care care dolphins compared to the free-ranging dolphins. Differences in immune parameters appear to reflect the environmental conditions under which these four dolphin populations live which vary widely in temperature, nutrition, veterinary care, pathogen/contaminant exposures, etc. Many of the differences found were consistent with reduced pathogenic antigenic stimulation in managed-care care dolphins compared to wild dolphins. Managed-care care dolphins had relatively low T_H_2 lymphocyte activity and fewer circulating eosinophils compared to wild dolphins. Both of these immunologic parameters are associated with exposure to helminth parasites which is uncommon in managed-care care dolphins. Less consistent trends were observed in a suite of hormones but significant differences were found for cortisol, ACTH, total T_4_, free T_3_, and epinephrine. While the underlying mechanisms are likely multiple and complex, the marked differences observed in the immune and endocrine systems of wild and managed-care care dolphins appear to be shaped by their environment.

## Introduction

Wild marine mammals are exposed to multiple natural and anthropogenic environmental stressors [1, 2], [3]. Physiological and pathologic responses to these environmental stressors play a critical role in allowing animals to cope with environmental perturbations, and are largely uncharacterized in marine mammals. There remains a large gap in our knowledge about the pathophysiological effects of both acute and chronic stress in marine mammals. Concerns about anthropogenic stressors faced by wild marine mammals include increased environmental exposures to pathogens, pollution, and noise [1]. Studies that evaluate these effects in wild marine mammals are lacking and baseline data related to marine mammal physiology and health are few. To manage wild marine mammal populations in light of growing anthropogenic stressors, studies are required to better understand baseline health metrics and the cumulative effects resulting from multiple stressors.

Although the basic physiology of the stress response is well understood in terrestrial mammal models, there remains much to be discovered about how the stress response has been modified by the aquatic evolution of marine mammals. A recent review of stress in marine mammals supports the terrestrial model as a useful foundation with several specific differences unique to marine mammals [4]. In marine mammals under human care, the stress response has been shown to adhere to the classic model of the general adaptation syndrome with activation of the hypothalamic-pituitary-adrenal (HPA) axis resulting in release of adrenocorticotropic hormone (ACTH) from the pituitary into the bloodstream, and secretion of cortisol and other corticosteroids from the adrenal cortex [5, 6]. The involvement of the sympathetic nervous system in the stress response is responsible for release of the catecholamines (e.g., epinephrine (EPI) [7]. Examination of corticosteroid hormones and catecholamines in wild-caught dolphins demonstrated that the general mammalian response to the acute stress of capture and restraint could be elicited [8].

Studies have shown differences in endocrine hormones between managed-care and wild marine mammals. For example, lower levels of thyroid hormones have been observed in managed-care pinnipeds, manatees and cetaceans compared with their wild counterparts [5, 9, 10]. However, knowledge about the immune systems of most marine mammal species remains fragmentary [11] and comparative studies between wild and managed-care populations are lacking. One recommendation to advance the knowledge of the impact environmental influences have in marine mammals is to obtain contextual data on the natural variation in a suite of hormones and influences on the mediators of endocrine responses that can be used for comparison [4]. In order to consider inherent differences between managed-care and wild animals, it is therefore critical to develop baseline measurements of multiple endocrine and immune markers in both types of populations. Here we report on a study that compares endocrine and immune markers from wild and managed-care dolphins, which are subject to a range of environmental conditions. We evaluate relationships between environmental conditions (wild vs. under human care) and immunological markers, dependent hormonal endpoints, and clinical pathology parameters.

## Methods

### Study populations

Samples were collected from four groups of bottlenose dolphins (*Tursiops truncatus*): 1) dolphins from the Georgia Aquarium (GA), which were under managed-care in a closed or semi-open water environment; 2) dolphins from the U.S Navy Marine Mammal Program (MMP), which were under managed-care and were in an open-water (bay) environment; 3) wild dolphins from the Indian River Lagoon, (IRL) FL; and 4) wild dolphins from Charleston, (CHS) SC.

### GA dolphins

Samples were obtained from 10 dolphins managed by the Georgia Aquarium at facilities in Atlanta, GA and at Marineland, FL. Three of the 10 GA dolphins sampled came from the wild and the remainder were born under human care. The Georgia Aquarium is a USDA APHIS approved and inspected facility and a member of Association of Zoos and Aquariums (AZA) and the Alliance of Marine Mammal Parks and Aquariums (AMMPA). The GA dolphin environment in Atlanta is in a controlled indoor environment with artificial sea water maintained at 24° C. The GA dolphin environment at Marineland is outdoors with controlled natural seawater temperatures that range from 24-29° C. Life support systems at both facilities include sand filtration, protein skimming and ozonation and/or chlorination. Samples were collected between September 2011 and August 2012. Blood samples were obtained from the arteriovenous plexus of the fluke using voluntary husbandry behaviors as part of a preventative medical program. Multiple collections were made during the 12-month period for a total of 37 samples (Table 1).

**Table 1:** Demographic data for bottlenose dolphins from managed-care (Marine Mammal Program (MMP), Georgia Aquarium (GA)) and wild populations (Indian River Lagoon (IRL), FL, Charleston (CHS), SC.).

	MMP	GA	IRL	CHS
Individuals	7	10	26	19
Blood Samples	45	37	26	19
Gender				
Male	3 (43%)	4 (40%)	15 (58%)	12 (63%)
Female	4 (57%)	6 (60%)	11 (42%)	7 (37%)
Mean Age (yrs) (±SD)	27.4 (8.5)	16.9 (8.9)	14.0 (7.0)	17.8 (7.5)
Age (range yrs)	9–41	8–28	6–25	3–33

### MMP dolphins

The MMP dolphin population resides at the Space and Naval Warfare Systems Center (SSC) Pacific located in San Diego, California. Dolphins live within open-water, netted enclosures within San Diego Bay and are exposed to ambient air and water temperatures throughout the year (15-22° C). All MMP dolphins have been maintained in and exposed to natural environmental conditions throughout their lives. Two out of 7 of the MMP dolphins were wild-caught over 20 years prior to the study, while all others were born at the MMP. All procedures conducted with MMP dolphins were approved by the Institutional Animal Care and Use Committee (IACUC) of the Biosciences Division, SSC Pacific and the Navy Bureau of Medicine and Surgery, and followed all U.S. Department of Defense guidelines for the care of laboratory animals. Blood samples were obtained from the arteriovenous plexus of the fluke by voluntary, trained behavior. Sampling was conducted during September 2011 to August 2012 with collection of 45 samples from 7 dolphins (Table 1).

### IRL and CHS dolphins

This group was comprised of populations of wild dolphins from two estuarine areas of the southeast Atlantic Ocean United States; Indian River Lagoon, FL and Charleston, SC as part of the Dolphin Health and Risk Assessment (HERA) Project, a multidisciplinary, integrated, collaborative effort to assess individual and population health. Detailed information pertaining to the study site, methods of capture, sampling and release are described elsewhere [12]. Once restrained, blood samples were drawn, generally within the first 10 min of capture, from the periarterial rete of the flukes. Age was determined by counting post-natal dentine layers in an extracted tooth [13]. During capture-release health assessments, samples were collected from 26 dolphins in the IRL in July 2011 and 19 dolphins in CHS in August 2013 (Table 1). Samples were collected under National Marine Fisheries Permit No. 14352–03 (permit dates from 2010–2015) issued to Dr. Gregory Bossart and approved by the Florida Atlantic IACUC under Protocol #A10-18. The health of dolphins in both IRL and CHS populations is considered compromised with fewer than half found to be clinically normal [14]. Multiple environmental stressors exist at each of the sites inhabited by the wild dolphins suggesting impacts on both populations, albeit of differing origins. Freshwater discharge and heavy metal contamination with limited tidal flushing characterizes the environment of the IRL [15]. Higher mercury levels have been documented among IRL dolphins compared to CHS dolphins [16, 17]. Both IRL and CHS dolphin populations have been reported to have orogenital neoplasia that impact immune function and that are associated with novel papillomavirus and herpesvirus infections [18–22]. Additionally, IRL dolphins have reported subclinical or clinical infections of cetacean morbillivirus, lacaziosis and chlamydiosis that result in various, often severe, immunologic perturbations [23–25]. CHS is a densely-populated city with a rapid growth rate and Charleston Harbor receives multiple inputs from industrial, urban and suburban sources with elevated levels of organic contaminants such as polychlorinated biphenyls, chlorinated pesticides, polyaromatic hydrocarbons and perfluoroalkyl substances [26, 27]. Compared to IRL dolphins, CHS dolphins were found to accumulate significantly higher levels of persistent organic contaminants [28–30], which were associated with land use in the CHS area [31, 32].

### Hematology and serum protein electrophoresis

Standardized sampling methods for hematology and serum protein electrophoresis as described for the Dolphin HERA Project were used [12]. Whole blood was collected in vacutainer tubes containing sodium heparin or ethylenediamine-tetraacetic acid (EDTA) as anticoagulants and without anticoagulants in 10 mL serum-separator vacutainer tubes (Becton Dickinson, Franklin Lakes, NJ). Samples for clinical chemistry were held for 30–40 minutes and centrifuged at 1233 g for 15 min. Fibrin clots were removed and the serum transferred to plastic tubes. Serum was transferred to cryovials and stored at -80°C until analyzed. Serum was subsequently sent to the Animal Health Diagnostic Laboratory (AHDL) at Cornell University (Ithaca, New York) along with EDTA tubes for standard clinical pathology tests. Hematology, serum protein electrophoresis (SPEP) and reproductive hormones (progesterone, estradiol, testosterone) were determined by AHDL. The results of these investigations in wild dolphins from CHS and IRL have been published previously [33, 34]. Briefly, white and red blood cell counts, hemoglobin, total platelets and other red blood cell parameters were determined using an automated analyzer (Bayer ADVIA 120, Bayer Diagnostics, Tarrytown, NY, USA). Differential leukocyte counts were performed by microscopic examination of modified Wright-Giemsa stained blood smears (Bayer Healthcare, Tarrytown, NY, USA). A microhematocrit tube was centrifuged for 5 min at 11,700 rpm, and the manual hematocrit was interpreted by visual inspection. Serum protein electrophoresis was performed on an automated analyzer (Rapid Electrophoresis, Helena Laboratories, Beaumont, TX, USA).

### Hormone analyses

ACTH, aldosterone, cortisol, and thyroid hormones were assayed by enzyme-immunoassay (EIA) or radio-immunoassay (RIA). ACTH was assayed by EIA using a commercially available EIA kit (Alpco, Salem, NH) as described and validated in Champagne et al [35]; the coefficient of variation (CV) between sample replicates was 4.0%. Aldosterone concentrations are generally low in dolphin serum samples. We therefore conducted a steroid extraction to increase the detectable quantity of aldosterone before the RIA. Aldosterone was extracted from 1 mL serum into an organic solvent by the sequential addition of 4 mL dichloromethane (Sigma-Aldrich Inc., St Louis MO), vortexing, and collecting the organic phase. The extraction was repeated three times, collecting the dichloromethane into a single test tube and then evaporating to dryness on a vacuum centrifuge. The dry samples were reconstituted in 500 μL of steroid free serum (Siemens, Inc.) before assaying using a RIA kit (Siemens Inc.) that has been previously validated for bottlenose dolphins [36]. We corrected the assay value by the dilution factor of the sample extraction to determine serum aldosterone concentration; the CV between sample replicates was 1.8%. We tested the extraction efficiency using tritiated aldosterone standards and found greater than 90% recovery; therefore, no correction for extraction efficiency was conducted. Aldosterone was not assessed in IRL dolphins due to insufficient sample volume. Cortisol was assayed using a RIA kit previously validated for use in bottlenose dolphin [36, 37]. Cortisol concentrations were similarly low in dolphin serum. We therefore modified the RIA protocol by increasing the recommended serum volume from 25 μL to 100 μL. We tested the validity of this modification by comparing cortisol standards assayed and found proportional increases up to 150 μL sample volume; sample cortisol concentrations were corrected for volume simply by dividing by four. The CV between sample replicates was 2.2%. Serum thyroid hormone concentrations, triiodothyronine and thyroxine (T_3_ and T_4_, respectively), were assessed using commercially available kits to detect free (unbound) and total (both unbound and bound with carrier protein) T_3_ and T_4_. Thyroid hormone assays were conducted according to the kit protocols (Siemens, Inc.) as validated by Ortiz et al. [37]. The replicate CV’s for thyroid hormones were 1.8, 2.6, 2.2 and 3.2% for free and total T_3_ and T_4_, respectively. Catecholamines were measured by Mystic Aquarium, (Mystic, CT) using methods detailed previously ([38]. Briefly, high performance liquid chromatography (HPLC) with electrochemical detection was used to measure and quantify dolphin catecholamines (EPI, DA) following the methodology detailed in the manufacturers (BioRad, Hercules, CA) manual on Plasma Catecholamines by HPLC. Quantitation of EPI and DA was based on comparing peak height ratios relative to an internal standard in both the unknown sample and a plasma calibrator specimen (BioRad plasma catecholamines by HPLC Calibrator, 195–6066; Siemens Medical Diagnostics, Los Angeles, CA). Data were analyzed using Breeze software (Waters, Milford, MA).

### Immune assays

Immunophenotyping (B and T cell lymphocyte subsets, and MHCII+ expression) were measured at Mystic Aquarium using methods previously described [22]. Briefly, 1 × 10^6^ cells/mL were labeled with 50 μL of monoclonal supernatant or purified antibody for 30 min at 4°C. This was followed by incubation with fluorescein isothiocyanate conjugated affinity purified goat anti-mouse F(ab)’2 IgG1 for 30 min at 4°C in the dark. Cells were analyzed using flow cytometry on an LSR flow cytometer (BD Biosciences, San Jose, CA, USA).

Measurements of natural killer cell and lysozyme activity were conducted in the NOAA Center for Coastal Environmental Health and Biomolecular Research Laboratory (Charleston, SC). Peripheral blood leukocytes (PBLs) were isolated from whole blood samples by a slow spin technique (700 rpm in a swinging bucket rotor for 25 min) within 36 h of blood collection. The PBLs were re-suspended in 1 ml of complete media (RPMI-1640, 10% fetal bovine serum, 50 IU penicillin and 50 mg streptomycin). Numbers of nucleated PBLs were determined using a hemocytometer following RBC lysis with zap-o-globin or 0.17 M ammonium chloride. Cells were then diluted as required. Natural killer (NK) cell activity was assessed via an *in vitro* cytotoxicity assay using 51Cr-labeled Yac-1 cells as previously detailed [22]. Serum lysozyme activity was assessed using slight modifications of a standard turbidity assay using a solution of *Micrococcus lysodeikticus* as described previously [22].

Immunological analysis of IgG and pathogen ELISAs were conducted at Clemson University (Clemson, SC). The concentration of IgG was determined in a sandwich capture ELISA using two monoclonal antibodies as previously described [39]. Specific antibody activities against select marine bacteria were determined in an ELISA system also described previously [39]. Cultures of the following bacteria were obtained from ATCC and sub-cultured: *Escherichia coli*, *Erysipelothrix rhusiopathiae*, *Mycobacterium marinum*, *Vibrio cholerae and Vibrio parahemolyticu*s.

A broad array of leukocyte cytokine and lymphocyte activation transcript levels was determined (IL4, IL10, IL17, TNFα, IFNg, IFNa, MX1, CD69 and IL2-Ra) at UC Davis, as previously described [40–42]. Blood samples were collected into vacutainers (PAXgene^TM^). Two markers are related to lymphocyte activation (CD69 and IL-2Rα). The cytokines measured consisted of two pro-inflammatory (TNFα, IL-17), two antiviral (MX-1, IFNα), a marker of T-helper type 1 (T_H_1) activity (IFNγ), and a marker of T-helper type 2 (T_H_2) activity (IL-4) and IL-10 which plays a regulatory function and has anti-inflammatory activity. Total RNA was extracted using a commercial kit and cDNA synthesized using a commercial reverse transcription kit (Qiagen, Valencia, CA). Real-time PCR analysis was conducted as follows. Briefly, 5 μL of diluted (1:5) cDNA was added to a mixture containing 12.5μl of QuantiTect SYBR Green^®^ Master Mix [5mM Mg^2+^] (Qiagen, Valencia, CA), 1μl each of forward and reverse sequence specific primers, 0.5μl of Uracil-N-Glycosylase (Invitrogen, Carlsbad, CA), and 5.0μl of RNase-free water. Samples were loaded into 96 well plates in duplicate and sealed with optical sealing tape (Applied Biosystems, Foster City, CA). Reaction mixtures containing water, but no cDNA, were used as negative controls. Amplifications were conducted on a 7300 Real-time Thermal Cycler (Applied Biosystems, Foster City, CA). Reaction conditions were as follows: 50°C for 2 minutes, 95°C for 15 minutes, 40 cycles of 94°C for 30 seconds, 55°C for 30 seconds, 72°C for 31 seconds, an extended elongation phase at 72°C for 10 minutes, followed by a dissociation step. Product specificity was monitored by analysis of melting curves and periodically confirmed by sequencing the amplified products. Threshold crossing values for cytokine genes of interest were normalized (NV) to the S-9 ribosomal gene; NV = Threshold crossing (TC) of gene of interest–TC of housekeeping gene. The smaller the normalized value, the more a gene was being transcribed; the larger the normalized value, the less a gene was transcribed.

### Data analyses

Descriptive statistical analysis for each population was conducted to calculate the mean and standard deviation for all test variables. Normality was assessed using the Shapiro-Wilk test and variables were log transformed to meet test assumptions. To compare mean values across study populations a nested General Linear model (GLM) was used. Study location and sex were entered as fixed effects, age was a covariate, and individuals were a random effect. Post-hoc comparisons of variable means between locations were conducted by Least Significant Difference Test. Statistical significance was established at p < 0.05. All analyses were conducted using SPSS version 22 (IBM Corp. 2011, Armonk, NY).

## Results

### Collection summary

Table 1 provides a summary of demographic data for the four groups of dolphins. A total of 127 samples were collected during the study period. Multiple samples were collected from each of the seven MMP dolphins (n = 45) as well as from the 10 Georgia Aquarium dolphins (n = 37) while single samples were obtained from both wild dolphin populations (IRL = 26 and CHS = 19). Samples were obtained from males and females for all groups with females constituting the largest proportion in the managed groups and males the highest proportions in the wild groups. Mean ages ranged from 14.0 yrs. for IRL dolphins, 16.9 yrs. for Georgia aquarium dolphins, 17.8 yrs. for CHS dolphins and 27.4 yrs. for MMP dolphins.

### Comparison of endocrine and immune markers in managed-care and wild dolphins

The results of statistical analysis for each group of parameters are presented in Tables 2–4. The overall significance of the inter-population comparisons is summarized as the GLM p-value. The post-hoc p-values presented for each of the inter-population comparisons can be used to assess differences between individual populations of dolphins for each parameter. Comparison of mean values between the managed and wild dolphin groups indicated significant differences in several hematological parameters (Table 2). Generally, significant differences were found between managed-care and wild populations; significant differences in these parameters were not found between the two wild dolphin populations but were present in comparisons between wild dolphins and one or more of the managed-care dolphin groups. For instance, managed-care dolphins had significantly lower red blood cell (RBC) packed cell volume (PCV), hematocrit (Hct), hemoglobin (Hb), mean corpuscular hemoglobin (MCH), and mean corpuscular volume (MCV) than CHS dolphins, and lower RBC and MCH concentration (MCHC) than IRL dolphins. Significantly higher total WBC concentrations were observed in the wild dolphin groups versus managed-care dolphins. Generally absolute numbers of lymphocytes, eosinophils and platelets were higher in wild dolphins compared to managed-care dolphins. The absolute neutrophil concentrations (10^3^ cells/μL) were higher in wild dolphins than in managed-care dolphins but the differences were not significantly different. Mean absolute lymphocyte values (10^3^ cells/ μL) were significantly higher in both wild dolphin populations, nearly twice that of both groups of managed-care dolphins. CHS dolphins also had significantly higher lymphocyte values than IRL dolphins. Marked significant, differences in the mean absolute number of eosinophils (10^3^ cells/μL) were observed among all groups in with lowest levels occurring in GA dolphins (0.2), followed by MMP (0.9), IRL (3.1) and CHS (5.8) dolphins. The GA dolphins had significantly different levels of most serum fractions measured by electrophoresis compared to wild dolphins (Table 3). GA dolphins had significantly lower levels of total protein due to lower total acute phase proteins [alpha globulins and beta globulins] and total gamma globulin fractions than wild dolphins.

**Table 2:** Nested general linear model, means, standard deviation (SD) for hematological parameters for bottlenose dolphins from managed-care (Marine Mammal Program (MMP) and Georgia Aquarium (GA)) and wild populations (Indian River Lagoon (IRL) and Charleston (CHS)), adjusted for age and sex, by site.

	MMP		GA		IRL		CHS		GLM p-value	MMP vs GA	MMP vs IRL	MMP vs CHS	GA vs IRL	GA vs CHS	IRL vs CHS
	Mean	SD	Mean	SD	Mean	SD	Mean	SD							
Packed Cell Volume	35.59	1.32	38.43	0.87	41.09	1.22	40.09	1.34	**<0.02**	**0.03**	**0.01**	**0.01**	0.11	0.32	0.67
Hematocrit (%)	37.02	1.01	40.36	0.77	42.02	1.10	46.02	1.17	**<0.01**	**<0.01**	**0.01**	**<0.01**	0.27	**<0.01**	0.06
Hemoglobin (Hb)(g/dl)	12.49	0.33	13.78	0.25	14.71	0.36	14.91	0.38	**<0.01**	**<0.01**	**<0.01**	**<0.01**	**0.06**	**0.02**	0.77
RBC (10^3^ cells/μl)	3.34	0.08	3.62	0.06	3.62	0.09	3.72	0.10	**0.01**	**<0.01**	0.06	**<0.01**	**<0.01**	0.42	0.57
Mean Corpuscular Volume (MCV) (fl)	111.87	1.64	112.11	1.26	115.78	1.79	125.87	1.90	**<0.01**	0.83	0.17	**<0.01**	.132	**<0.01**	**0.01**
MC Hemoglobin (MCH) (pg)	37.86	0.52	38.46	0.40	40.79	0.57	40.46	0.60	**<0.01**	0.22	**<0.01**	**<0.01**	**0.01**	**0.01**	0.76
MCHC Conc. (g/dl)	33.47	0.44	33.80	0.34	35.13	0.48	32.47	0.51	**0.04**	0.42	**0.03**	0.11	**0.04**	**0.04**	**0.01**
WBCs (10^3^ cells/μl)	6.07	0.89	5.73	0.68	12.46	0.97	11.03	1.03	**<0.01**	0.70	**<0.01**	**<0.01**	**<0.01**	**<0.01**	0.44
Segmented Neutrophils (10^3^ cells/μl)	3.99	0.81	4.45	0.62	5.48	0.88	4.15	0.94	0.78	0.55	0.30	0.80	0.39	0.80	0.43
Band Neutrophils (10^3^ cells/μl)	1.00	0.48	1.07	0.48	1.95	0.87	2.17	1.01	**<0.01**	1.00	**0.03**	**0.01**	**0.01**	**<0.01**	0.82
Lymphocytes (10^3^ cells/μl)	1.11	0.04	1.00	0.05	1.95	0.05	2.17	0.06	**<0.01**	0.09	**<0.01**	**<0.01**	**<0.01**	**<0.01**	**0.01**
Monocytes (10^3^cells/μl)	0.25	0.15	0.32	0.12	0.13	0.17	0.39	0.18	0.83	0.61	0.83	0.52	0.56	0.77	0.54
Eosinophils(10^3^cells/μl)	0.88	0.29	0.24	0.22	3.08	0.31	5.78	0.33	**<0.01**	0.02	**<0.01**	**<0.01**	**0.01**	**<0.01**	**<0.01**
Basophils (10^3^ cells/μl)	0.06	0.02	0.10	0.02	0.06	0.03	0.06	0.03	0.39	0.15	1.00	**<0.01**	0.37	0.37	**<0.01**
Platelets (10^3^/μl)	134.69	10.22	97.69	7.81	192.36	11.12	158.69	11.82	**<0.01**	<0.01	**<0.01**	0.10	**<0.01**	**<0.01**	0.12

**Table 3:** Nested general linear model, means, standard deviation (SD) for protein electrophoresis for bottlenose dolphins from managed-care Georgia Aquarium (GA) and wild populations (Indian River Lagoon (IRL), FL, Charleston (CHS), SC, adjusted for age and sex, by site.

	Units	GA		IRL		CHS		GLM p-value	GA vs IRL	GA vs CHS	IRL vs CHS
		Mean	SD	Mean	SD	Mean	SD				
**PROTEIN ELECTROPHORESIS**											
Total Protein	g/dl	6.27	0.14	7.18	0.12	7.32	0.10	**<0.01**	**<0.01**	**<0.01**	1.00
Albumin	g/dl	3.68	0.09	3.26	0.08	3.29	0.07	**<0.01**	**0.01**	**<0.01**	**0.04**
Globulin	g/dl	1.54	0.28	2.78	0.50	2.99	0.65	**<0.01**	**<0.01**	**<0.01**	0.41
Albumin/Globulin ratio	g/dl	3.25	0.69	1.65	0.32	1.49	0.42	**<0.01**	**<0.01**	**<0.01**	0.45
Alpha1 Globulin	g/dl	0.22	0.02	0.28	0.02	0.28	0.01	**<0.01**	**<0.01**	0.05	0.25
Alpha2 Globulin	g/dl	0.90	0.04	0.99	0.03	1.21	0.03	**<0.01**	0.17	**<0.01**	**<0.01**
Total Alpha Globulins	g/dl	1.13	0.04	1.26	0.04	1.49	0.03	**<0.01**	**<0.01**	**<0.01**	**<0.01**
Beta1 Globulin*	g/dl	0.16	0.02	0.21	0.01	0.22	0.01	**0.01**	**0.01**	**0.02**	0.91
Beta2 Globulin	g/dl	0.21	0.03	0.25	0.03	0.27	0.02	0.15	0.55	0.14	0.45
Total Beta Globulins*	g/dl	0.37	0.04	0.46	0.03	0.49	0.03	**0.03**	0.10	**0.03**	0.70
Gamma Globulins	g/dl	1.11	0.18	2.21	0.16	2.05	0.14	**<0.01**	**<0.01**	**<0.01**	0.28

N/A = not available; Bold = p<0.05

* Log10 used

**Table 4:** Nested general linear model, means, standard deviation (SD) for endocrine and catecholamine parameters for bottlenose dolphins from managed-care (Marine Mammal Program (MMP), Georgia Aquarium (GA) and wild populations (Indian River Lagoon (IRL), FL, Charleston (CHS), SC, adjusted for age and sex, by site.

	MMP		GAI		IRL		CHS		GLM p-value	MMP vs GA	MMP vs IRL	MMP vs CHS	GA vs IRL	GA vs CHS	IRL vs CHS
	Mean	SD	Mean	SD	Mean	SD	Mean	SD							
ACTH (pg/mL)	24.29	2.70	51.30	2.61	163.23	3.62	35.52	3.19	**<0.01**	**<0.01**	**<0.01**	0.25	**<0.01**	**<0.01**	**<0.01**
Cortisol (μg/dL)	0.84	0.34	0.66	0.33	1.33	0.47	0.67	0.42	**0.05**	0.66	0.38	0.77	0.32	0.98	0.41
Estradiol (pg/mL)	N/A	.	12.84	2.56	22.20	1.42	20.33	1.21	**0.01**	.	.	.	**<0.01**	**0.01**	0.32
Progesterone (ng/mL)	N/A	.	0.03	2.92	0.11	1.62	6.20	1.37	**0.04**	.	.	.	0.45	**0.04**	**0.04**
Testosterone (ng/mL)	N/A	.	11.35	4.73	7.81	1.68	4.54	1.41	0.22	.	.	.	0.53	0.21	0.15
Total T4 (ug/dL)	13.07	0.74	12.95	0.71	12.69	0.95	7.26	0.87	**0.03**	0.89	0.75	**<0.01**	0.85	**<0.01**	**<0.01**
Total T3 (ng/dL)	99.37	6.81	64.61	6.58	87.20	8.79	52.81	7.98	0.08	**<0.01**	0.26	0.09	0.07	0.32	**0.02**
Free T4 (ng/dL)	1.35	0.15	1.26	0.14	1.61	0.19	1.26	0.17	0.64	0.61	0.28	0.74	0.21	0.99	0.29
Free T3 (pg/mL)	1.76	0.25	0.94	0.45	1.31	0.39	1.68	0.31	**0.02**	0.23	0.47	0.88	0.13	< 0.01	0.15
Aldosterone (pg/mL)	15.25	7.60	15.66	8.30	N/A	N/A	25.66	4.65	0.32	0.94	.	0.15		0.18	.
Dopamine (pg/mL)	51.85	8.35	61.42	11.25	31.18	10.35	46.58	25.77	0.24	0.49	0.13	0.85	0.05	0.60	0.58
Epinephrine (pg/mL)	56.21	40.20	104.57	38.65	108.39	34.78	219.12	29.71	**0.04**	**0.05**	0.03	**<0.01**	0.80	**<0.01**	**0.01**

N/A = not available; Bold = p<0.05

As shown in Table 4, there was a significant difference in ACTH concentrations between the groups. Higher ACTH levels were observed in IRL dolphins (163.2 pg/mL) followed by GA dolphins (51.3 pg/mL), CHS dolphins (35.5 pg/mL), and MMP dolphins (24.3 pg/mL). The ACTH levels of IRL dolphins were significantly higher than all other dolphin groups; CHS dolphins were significantly higher than the MMP dolphins. The overall comparison of cortisol levels was of marginal statistical significance (p = 0.05). The highest cortisol levels were found in the IRL dolphins but these were not significantly different from any of the other dolphin groups. Epinephrine concentrations were significantly higher in wild dolphins compared to one or both managed-care groups; CHS dolphins had significantly higher levels of EPI versus IRL dolphins, as well.

There was a significant difference in total T_4_ between the groups. Mean total T_4_ levels were significantly lower in CHS dolphins (7.36 μg/dL) compared to all other groups (12.69, 12.95, and 13.07 μg/mL in IRL, GA, and MMP, respectively). No significant differences were found either in total T_3_ or free T_4_. There was a significant difference in the concentration of free T_3,_ the metabolically active form of thyroid hormone, between the groups. The free T_3_ concentration in GA dolphins was significantly lower than that in CHS dolphins.

A comparison of mean values between the managed dolphin groups and the wild dolphins indicated significant differences in numerous immune parameters (Table 5). Significant differences were found for MHCII+ cells, CD2+ T cells, CD4+ helper T cells and mature CD21 B cells across the four population groups. In general, managed-care dolphins had significantly lower absolute numbers of these parameters than wild dolphins. Serum IgG levels were significantly lower in both managed-care dolphin groups compared to the wild dolphin groups. Overall higher NK cell activity was found in the wild dolphins but significant differences were only between CHS and GA and MMP and IRL groups. Significantly higher IgG levels (mg/mL) were observed in the two wild dolphin populations (15.49 CHS and 13.90 IRL, followed by MMP 10.39 and GA 3.52). There were no significant differences observed between the two managed-care dolphin groups (MMP, GA) in absolute numbers of MHCII+ cells, CD4+ helper T cells and NK cell activity. Among the four groups, the GA dolphins had the lowest CD21 B cells-mature, serum IgG and lysozyme concentrations.

**Table 5:** Nested general linear model, means, standard deviation (SD) for immunological parameters for bottlenose dolphins from managed-care (Marine Mammal Program (MMP), Georgia Aquarium (GA)) and wild populations (Indian River Lagoon (IRL), FL, Charleston (CHS), SC, adjusted for age and sex, by site.

	MMP		GAI		IRL		CHS		GLM p-value	MMP vs GA	MMP vs IRL	MMP vs CHS	GA vs IRL	GA vs CHS	IRL vs CHS
	Mean	SD	Mean	SD	Mean	SD	Mean	SD							
MHCII+ (Abs Nos.)	1146.37	152.64	628.75	289.63	1767.58	241.57	1740.90	233.33	**0.01**	0.05	**0.02**	**0.01**	**<0.01**	**0.01**	0.94
CD2 T Cells (Abs Nos.)	518.43	85.01	421.49	154.67	1116.16	129.00	1105.21	124.61	**<0.01**	0.59	**<0.01**	**<0.01**	**<0.01**	**<0.01**	0.95
CD4 Helper T Cells (Abs Nos.)	288.25	59.04	199.82	107.94	781.27	90.03	602.85	86.96	**<0.01**	0.50	**<0.01**	**<0.01**	**<0.01**	**0.01**	0.15
CD21 B Cells-mature (Abs Nos.)	524.45	72.15	189.41	139.66	391.51	116.48	504.62	112.51	0.21	0.05	0.48	0.98	0.26	0.08	0.48
Lysozyme Conc. (μg/μl)	10.95	1.38	3.20	1.53	12.38	0.98	7.83	1.19	**0.01**	**<0.01**	**0.02**	0.13	**<0.01**	**0.02**	**<0.01**
IgG1 (mg/ml)	10.51	0.29	11.69	0.47	13.90	0.67	15.49	0.57	**<0.01**	**0.04**	**<0.01**	**<0.01**	**<0.01**	**<0.01**	0.07
Natural Killer Cells (100:1)	5.37	3.89	3.52	4.21	18.71	3.98	10.46	3.58	**0.04**	0.76	**0.03**	0.37	**0.01**	0.21	0.12
**ANTIBODY TITERS** (μg/μL)															
*Mycobacterium marinarum*	158.79	8.35	119.05	19.84	190.80	16.53	122.96	14.12	**0.01**	0.19	0.22	0.06	**0.01**	0.87	**<0.01**
*Erysipelothrix rhusiopathiae*	147.59	7.05	73.11	15.51	197.10	12.91	221.66	11.04	**<0.01**	0.05	**0.01**	**<0.01**	**<0.01**	**<0.01**	**0.02**
*Vibrio cholera*	323.40	14.36	202.91	24.00	396.89	19.99	287.05	17.08	**<0.01**	**<0.01**	0.08	0.11	**<0.01**	**0.01**	**<0.01**
*Escherichia coli*	153.32	11.10	123.76	18.41	221.43	23.27	269.39	17.16	**<0.01**	0.18	**<0.01**	**<0.01**	**<0.01**	**<0.01**	**0.04**
*Vibrio parahaemolyticus*	132.94	6.79	160.46	11.15	181.86	11.27	201.76	10.50	**<0.01**	**0.04**	**<0.01**	**<0.01**	0.17	**0.01**	0.17
**CYTOKINE EXPRESSION**															
TNF	13.19	1.05	12.70	0.62	8.94	2.35	10.45	1.21	**<0.01**	0.74	**0.01**	0.20	0.18	**0.03**	0.67
IL-17	15.96	3.12	18.05	1.85	12.60	6.96	13.61	3.59	0.13	0.67	0.47	0.71	0.51	0.16	0.92
MX1	19.02	3.97	16.85	2.35	8.81	8.84	12.89	4.56	0.20	0.70	0.08	0.45	0.45	0.39	0.76
INFα	9.98	1.27	10.60	0.75	6.03	2.83	8.74	1.46	**0.01**	0.73	**0.04**	0.63	0.18	0.15	0.52
IL-4	12.46	3.84	21.85	4.85	17.79	4.74	16.15	2.79	**.011**	0.28	0.53	0.57	**0.01**	**0.03**	0.50
IL-10	10.97	1.04	11.05	0.62	8.04	2.32	8.59	1.20	**<0.01**	0.96	0.06	0.27	0.28	**0.02**	0.87
CD69	10.30	0.66	10.38	1.41	7.35	1.97	N/A	N/A	0.08	0.97	0.11	.	0.37	.	.
IRL2Rα	13.90	0.85	12.41	0.50	12.00	1.90	9.49	0.98	**<0.01**	0.22	0.13	**0.01**	0.86	**<0.01**	0.38
INFγ	13.50	1.26	15.12	0.75	11.60	2.81	14.41	1.45	0.38	0.37	0.31	0.72	0.30	0.58	0.50

N/A = not available; Bold = p<0.05

**Fig 1:**
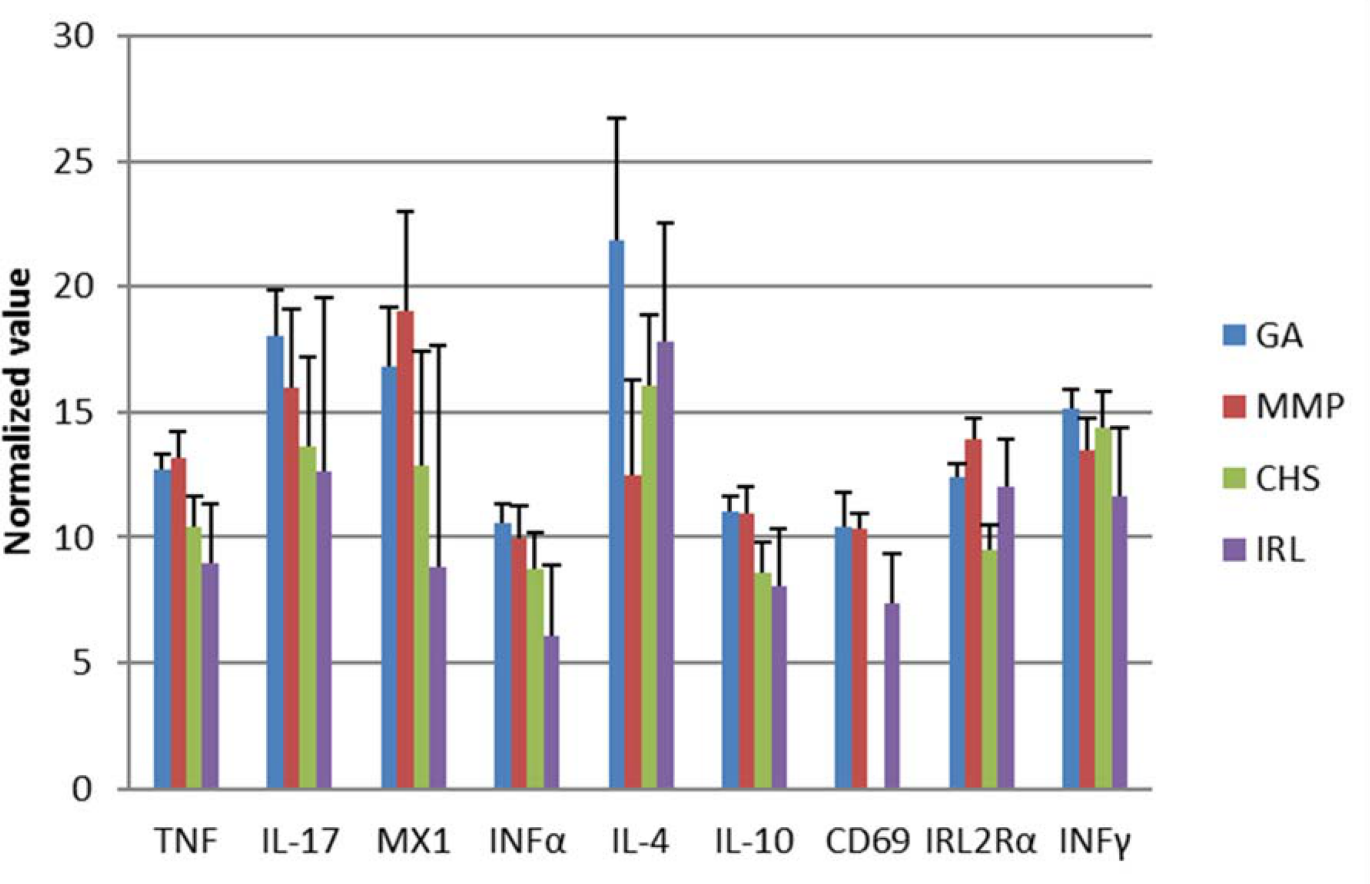
Gene transcript levels of measured cytokines from managed-care (Navy, Georgia Aquarium (GA)) and wild populations (Indian River Lagoon (IRL), FL, Charleston (CHS), SC), adjusted for age and sex, using a general linear model. Statistical differences between groups were found for the following five cytokines TNF, INFα, IL-4, IL-10, IRL2Rα (see Table 5 for statistical differences). Generally, higher transcript levels were found in wild dolphins versus one or both managed-care groups (note, the smaller the normalized value, the more a gene is transcribed, the larger the normalized value, the less a gene is transcribed).

We incorporated a suite of 7 cytokines and 2 lymphocyte activation markers (IL4, IL10, IL17, TNFα, IFNg, IFNα, MX1, CD69 and IL2-Rα) in the immunological analysis of samples collected from the dolphin groups. As shown in Table 5 and Fig 1, GA and MMP dolphins had significantly lower levels of transcripts encoding the two pro-inflammatory cytokines IL-17 and TNF, anti-viral MX1 and INFα, and regulatory IL-10. IL-2Rα and CD69 (not measured in the MMP dolphins), markers associated with lymphocyte activation, were lowest in free ranging dolphins. IL-4, a cytokine associated with T_H_2 activity, was significantly lower in GA dolphins compared to the free-ranging animals. Generally, the GA dolphins had the lowest transcript levels for all parameters measured, although the magnitude of these differences was sometimes small.

**Fig 2:**
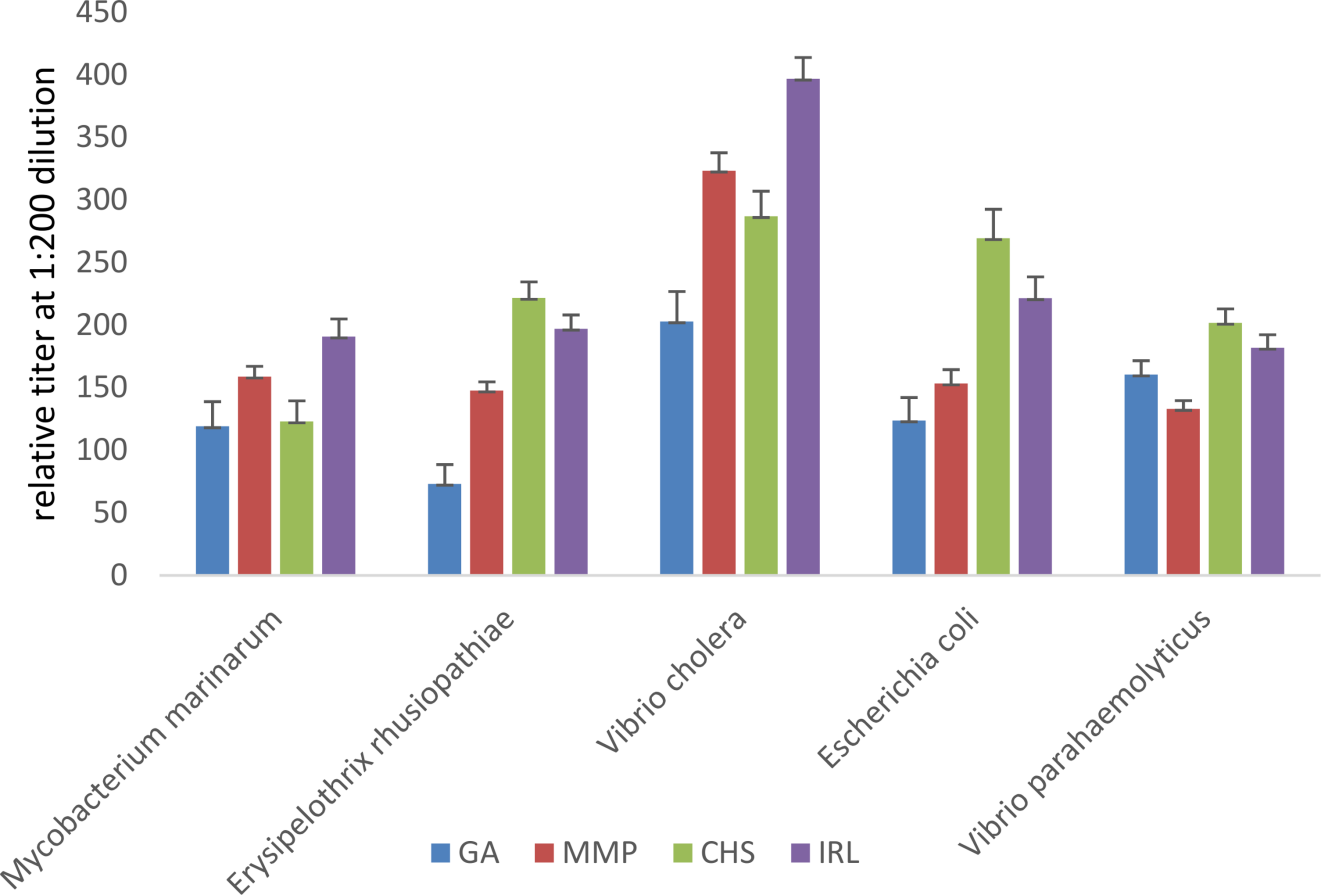
Means and standard deviation of antibody titers to marine bacteria from managed-care (Navy, Georgia Aquarium (GA)) and wild populations (Indian River Lagoon (IRL), FL, Charleston (CHS), SC), adjusted for age and sex, using a general linear model. For all antibodies, significantly higher titers were found in wild dolphins compared to one or more of the managed dolphin groups (see Table 5 for statistical differences). No differences were observed between the two managed-care dolphin groups with their antibody titers to V. cholera and *M*. *marinarum* but differences occurred in the other three organisms (*E*. *rhusiopathiae*, *V*. *parahaemolyticus*, *E*.*coli*).

Antibody titers against several common marine bacteria (*E*. *coli*, *E*. *rhusiopathiae*, *V*. *cholera*, *V*. *parahaemolyticus*, *M*. *marinarum*) were measured as an indication of the humoral immune response. Antibody titers were significantly higher in wild dolphins compared to one or both of the managed-care dolphin groups (Table 5 and Fig 2). Significantly higher titers to *E*. *rhusiopathiae*, *V*. *parahaemolyticus*, *and M*. *marinarum* were found in MMP dolphins, living in an open bay environment, compared to the GA dolphins maintained in treated water.

## Discussion

The results of our study suggest that the immune systems of wild dolphins are more upregulated than those of managed-care dolphins. This is evident in higher numbers of circulating lymphocytes and eosinophils, higher concentrations of gamma globulins and IgG, and higher levels of multiple immune components (i.e., MHCII, CD2, CD4 positive cells, lysozyme, NK cell function, and pathogen antibody titers). Both managed-care dolphins had significantly lower absolute numbers of MHCII+ cells and CD4+ helper T cells compared to wild dolphins. Additionally, GA dolphins had significantly lower levels of CD2+ T cells and mature CD21 B cells compared to wild dolphins. The relatively large differences between wild and managed-care dolphins in these parameters suggest that the environment exerts an important influence on immune system activity.

Managed-care dolphins had significantly lower serum IgG and NK cell activity compared to the wild dolphin groups. IgG binds pathogens and toxins and has an important role in antibody-dependent cell-mediated cytotoxicity. Natural killer (NK) cells play an important role in innate immunity by lysing and killing nonself/foreign cells, tumor cells and virus-infected cells [43]. Their activity is another commonly measured functional component of the innate immune response [43, 44]. Although the two-managed care dolphin groups (MMP, GA) differed in closed/ semi-open vs open systems, there were no significant differences in their absolute numbers of MHCII+ cells, CD4+ helper T cells, CD21 B cells-mature and NK cell and lysozyme activity. The GA dolphins had the lowest concentrations of CD21 mature B cells, serum IgG, and lysozyme among the dolphin groups, consistent with lower exposures to pathogens and foreign antigens. Along these same lines, the lower levels of acute phase proteins (total alpha globulins and total beta globulins) in GA dolphins likely reflect the absence of acute systemic inflammation, which is also consistent with lower exposure to foreign pathogenic antigens [45].

Antibody titers to common marine organisms were significantly higher in wild dolphins compared to one or both of the managed dolphin groups. The differences in antibody concentrations suggest that managed-care dolphins have reduced antigenic exposure to infectious agents. Similar antibody titers to *V*. *cholera* and *E*.*coli* were found in both managed-care dolphin groups. However, the antibody titers to *E*. *rhusiopathiae*, *V*. *parahaemolyticus*, and *M*. *marinarum* were higher in the MMP dolphins likely reflecting exposure to these microbes in open-water pens.

Selected cytokine gene transcript levels were determined, as were selected markers of lymphocyte activity, CD69 (not measured in MMP dolphins) and IL-2Rα. Cytokines are low molecular-weight regulatory proteins or glycoproteins that serve as soluble messengers of the immune system. They are differentially produced by leukocytes, and various other cells, and play a pivotal role in leukocyte development, response, effector function, traffic, inflammation, etc. While the number of gene transcripts measured in the current study was relatively small, they were representative of pro-inflammatory responses (TNFα), T_H_1 responses associated with clearance of intracellular pathogens (IFNγ), T_H_2 responses associated with helminthic infections and allergy (IL-4), anti-viral responses (MX1 & IFNα), T lymphocyte activation (CD69 and IL2Rα) and regulatory responses required to provide negative feedback on immune responses (IL-10). The above-mentioned cytokine activities are often pleomorphic in their activity and as such, can play additional roles other than those mentioned. Examination of the data (Table 3) demonstrates relative differences in transcript levels between wild and managed-care populations for most genes studied; the relatively higher levels in wild populations are consistent with greater microbial or foreign pathogenic antigen insults. Differences in transcript levels of IL-4 were quite dramatic. IL-4 is the hallmark product of T_H_2 lymphocytes and is associated with production of IgE and elimination of helminthic parasites, which wild dolphins would be expected to harbor routinely. The managed-care dolphins receive fish which are frozen, thus the parasites are non-viable and would be expected to carry a negligible or no load of these parasites. While the MMP dolphins may be exposed to these parasites in open water pens, they receive antihelminthics as part of their veterinary care. Interestingly, eosinophils, which are also associated with helminthic insults, were substantially higher in wild dolphins relative to their managed-care counterparts. Although IL-4 was high in the MMP dolphins, it was not statistically different from the wild dolphins and since both the MMP and wild dolphins are exposed to natural seawater this may account for their higher transcript levels compared to GA dolphins which are maintained in treated water.

The marked differences found in the overall level of activation of the cells of the immune system between wild and managed-care dolphins are not surprising given the differences in the environments in which they live. In contrast to wild dolphins, those in managed facilities are provided protection from predators, reliable and predictable food that has been inspected prior to consumption, and veterinary care. Closed system managed facilities are cleaner and controlled providing optimal water quality and a more stable environment along with implementation of pathogen control programs. Thus, data from the MMP managed-care population distinguishes it as an ‘in-between’ group, a hybrid, that live in open environments so they are exposed to waters that contain bacteria and viruses but also receive veterinary care and husbandry. Differences between managed-care and wild dolphins also include social structure and conspecific interactions. Wild dolphins must forage for their food, avoid predators and are exposed to a wider array of pathogens, contaminants and toxins.

Anthropogenic contaminants are one of many variables that can impact the immune system. The persistent organic contaminants (POCs), specifically polychlorinated biphenyl (PCBs) and the dichlorodiphenyltrichloroethane (DDT) family of pesticides have been shown to reduce the immune response in bottlenose dolphins and other cetaceans [46,47] (Lahvis et al., 1995; Schwacke et al., 2012). Generally, immune suppression is associated with exposure to POCs and enhanced vulnerability to infectious agents occurs with PCBs [48] (Hall et al., 2006). However, our studies have shown that the poly- and perfluoroalkyl substances (PFAS), which are elevated in the Charleston dolphins, have a pro-stimulatory effect both *in vivo* and *in vitro* [12, 49] (Fair et al.,2013; Soloff et al., 2017). Contaminant burdens were measured in IRL and CHS dolphins in several earlier studies. Generally, Charleston dolphins had higher POC concentrations [30] but IRL dolphins had higher levels of mercury [16,17]. We were not able to measure contaminant levels in the managed-care dolphin groups in this study to compare concentrations with the wild dolphins. A previous study by Reddy et al. [50] measured POCs in managed-care dolphins from the Marine Mammal Program. However, these data are not relevant to our current analysis, since the environmental concentrations, prey concentrations and sources of fish fed are not related to current conditions.

While the differences may seem intuitive, few studies have examined immune function in wild animals living in their natural environment, and fewer have compared immune function between wild and managed-care conspecifics. Compared to laboratory-bred mice, wild mice had higher concentrations and more avid antigen-specific IgG responses, as well as higher concentrations of total IgG and IgE [51]. Similarly, one study found that wild hyenas have significantly higher serum antibody concentrations, including total IgG and IgM, natural antibodies, and autoantibodies than do managed-care hyenas [52]. Different immune strategies are suggested to account for pathogen pressure for downregulated immune function exhibited in managed-care shorebirds, *Calidris canutus*, compared to free-living shorebirds [53]. Thus, it is expected that exposure to pathogens is likely to be a driver in our study with the GA dolphins exhibiting low values for many of the parameters measured (i.e., antibody titers, IgG, cytokine transcript levels). The GA dolphins are maintained in treated water systems. A closed man-made filtered aquatic system also provides a more hygienic environment limiting exposure to infectious agents, symbiotic microorganisms, and parasites. While decreased exposures may lead to a relatively quiescent immune system, previous observations in similar artificial environments demonstrated dolphins were capable of mounting a rapid and protective immune response to infectious agents and vaccines [9,54]. The MMP dolphins live in open floating pens in San Diego Bay and reflect an intermediate group between dolphins in closed system facilities and those living in the wild. As such, pathogen exposure would be greater for these animals compared to those in controlled, closed systems.

Assessing the physiological response to stress in wild dolphins is difficult since the processes of capture and blood collection induce stress [4, 8]. Therefore, a cautionary approach should be taken when evaluating these measurements. ACTH, thyroid hormone, aldosterone and catecholamine measurements varied significantly between the dolphin groups. In addition, the data for wild dolphins represent a single observation and may not adequately reflect baseline values, especially for stress-related parameters affected by the capture technique itself. Sampling procedures for blood collections from managed dolphins occurred via voluntary husbandry behaviors as indicated in the methods section. Both the environment and capture/handling responses may exert additive effects and need to be considered in the interpretation of results, particularly in hormones that are immediately released.

There was an overall difference in the concentration of ACTH across the four groups of dolphins. The highest levels of ACTH were found in the IRL dolphins which were significantly higher than each of the other groups. The differences in ACTH concentrations between the two wild dolphin populations is consistent with a recent study of acute stress during capture-release in which CHS dolphins had lower ACTH values than IRL dolphins [8]. The cortisol values for both wild dolphin populations measured during previous capture-release studies [8] were higher than those obtained in the present study. However, the earlier analyses for cortisol and ACTH were performed in another laboratory and methodological differences between laboratories may explain these differences. For aldosterone, CHS wild dolphins had the highest mean levels, nearly twice those of MMP and GA dolphins, although the linear model was not significantly different. Concentrations of aldosterone have been previously reported to be greater in managed-care dolphins versus wild dolphins [55, 56].

Significantly higher concentrations of EPI were found in CHS dolphins compared to all other groups (Table 3). Since blood was collected by voluntary husbandry techniques in both managed-care dolphins, it might be expected based upon terrestrial mammal models that EPI levels would be lower in both groups. However, EPI concentrations were similar in managed-care groups and the IRL dolphins. Only the CHS dolphins had significantly higher EPI concentrations in inter-population comparisons. This is consistent with a previous study on capture stress that also found the CHS dolphins had higher EPI values than the IRL dolphins, despite the use of similar techniques [8]. No differences were observed in dopamine levels for any of the groups.

Limited information exists on a comprehensive evaluation of stress in marine mammals and the relationship between hormones of the HPA axis and related hormones such as those produced by the thyroid gland. The latter play a critical role in numerous metabolic and physiological processes including regulation of basal metabolic rate, oxygen consumption, and carbohydrate and lipid metabolism [57–59]. Evidence predominately from laboratory and domestic animal studies also indicates thyroid hormone involvement in immune responses to environmental and stress-mediated suppression [60, 61]. A growing body of evidence supports the bidirectional communication between the neuroendocrine and immune systems [62, 63] specifically in cetaceans [38, 64, 65]. Our previous study showed that thyroid hormone concentration varies with age group and sex [66], thus, our analyses were adjusted for these covariates. Significant differences were found in total T_4_ and free T_3_ levels among the dolphin populations. For T_3_, GA dolphins had significantly lower levels compared to CHS dolphins. For total T_4_, significant lower levels were not only found between CHS dolphins and both managed-care groups but also between the other wild population, the IRL dolphins. No clear pattern was found for total T_3_ and free T_4._ Since there were no consistent patterns between wild and managed-care groups, other variables such season, and thus water temperature, likely influence this hormone and highlight consideration underlying mechanisms involved in the stress responses. As previously, described, higher levels of thyroid hormones in CHS compared to IRL dolphins may constitute an adaptive phenomenon to their colder environment [66]. Other biological and environmental variables, including contaminants such as polybrominated diphenyl ethers which are found in high concentrations in CHS dolphins [28] and are known to affect thyroid metabolism [67], may also contribute to the observed differences. As a complement to endocrine and immune system evaluations, we measured hematological and serum electrophoresis parameters. There have been limited studies comparing clinicopathologic parameters between managed dolphins and wild dolphins. Wild dolphins were reported by Asper et al. [68] to have significantly higher WBC, lower neutrophils and a higher percentage of eosinophils than managed-care dolphins. The present study found similar results for WBC and eosinophils while differences in neutrophil levels were only found between the two managed groups. Lower WBC and eosinophil levels in the managed-care populations may be associated with reduced exposure to infectious agents and parasitism, consistent with observations by Asper et al. [68].

## Conclusions

Using a complex suite of endocrine and immune parameters, the cross-population comparisons assessed in this study provide insight into the differences in immune and endocrine parameters between groups of dolphins living under disparate environmental conditions. Overall, the differences found in immune parameters appear to reflect differences in the environmental conditions under which these four dolphin populations live reflective of their exposures to pathogens and foreign antigens. As expected, the largest differences were found between managed-care groups and wild dolphins reflective of the different environments in which they live and the veterinary care and diet supplied under managed-care conditions. The major differences occurred in the immune measurements. The findings related to the endocrine system were less easy to interpret since environmental variables such as water temperature and collection methods, factors that influence -measured hormones, varied between the populations.

Collectively, these data indicate that the environment shapes the immune and endocrine responses in dolphins. Many of the differences found between managed-care dolphins and wild dolphins are consistent with their environments and associated pathogenic antigenic stimulation. On the other hand, the biological significance of these differences cannot be determined; many of the statistically significant differences may not be accompanied by a biologically significant response or health effect. A more robust understanding of the effects of environmental variables such as pathogen exposure on immune function may be obtained by further studies assessing how basic immune defenses differ between wild and managed-care animals. A greater understanding of the interplay between the dolphins’ endocrine and immune systems and the role of such interactions in clinically relevant responses may provide valuable insights into how disruption within one or more of these compartments may influence the dolphins’ ability to regulate inflammation and disease processes.
